# Alcohol Abuse Associated With Increased Risk of Angiographic Vasospasm and Delayed Cerebral Ischemia in Patients With Aneurysmal Subarachnoid Hemorrhage Requiring Mechanical Ventilation

**DOI:** 10.3389/fcvm.2022.825890

**Published:** 2022-05-10

**Authors:** Lei Zhao, Chao Cheng, Liwei Peng, Wei Zuo, Dong Xiong, Lei Zhang, Zilong Mao, Jin'an Zhang, Xia Wu, Xue Jiang, Peng Wang, Weixin Li

**Affiliations:** ^1^Department of Neurosurgery, Tangdu Hospital, The Fourth Military Medical University, Xi'an, China; ^2^Department of Public Health, School of Health Sciences and Practice, New York Medical College, Valhalla, NY, United States

**Keywords:** aneurysmal subarachnoid hemorrhage, alcohol abuse, retrospective study, clinical outcomes, angiographic vasospasm, delayed cerebral ischemia

## Abstract

**Objective:**

Although alcohol abuse has been indicated to cause cerebral aneurysm development and rupture, there is limited data on the impact of alcohol abuse on outcomes after an aneurysmal subarachnoid hemorrhage (aSAH). This study aims to investigate whether alcohol abuse increases the risk of angiographic vasospasm and delayed cerebral ischemia (DCI) in critically ill patients with aSAH.

**Methods:**

We conducted a secondary analysis based on a retrospective study in a French university hospital intensive care unit (ICU). Patients with aSAH requiring mechanical ventilation hospitalized between 2010 and 2015 were included. Patients were segregated according to alcohol abuse (yes or no). Multivariable logistic regression analysis was used to identify the independent risk factors associated with angiographic vasospasm and DCI.

**Results:**

The patient proportion of alcohol abuse was dramatically greater in males than that in females (*p* < 0.001). The Simplified Acute Physiology Score II (SAPSII) score on admission did not show a statistical difference. Neither did the World Federation of Neurosurgical Societies (WFNS) and Fisher scores. Patients with alcohol abuse were more likely to develop angiographic vasospasm (OR 3.65, 95% CI 1.17–11.39; *p* = 0.0260) and DCI (OR 3.53, 95% CI 1.13–10.97; *p* = 0.0294) as evidenced by multivariable logistic regression analysis.

**Conclusions:**

In this study, patients with alcohol abuse are at higher odds of angiographic vasospasm and DCI, which are related to poor prognosis following aSAH. These findings are important for the prevention and clinical management of aSAH.

## Introduction

Alcohol abuse is associated with an increased risk of death and cardiovascular disease (CVD). Excessive alcohol intake is one of the top three leading causes of premature deaths in the US (the other two are smoking and obesity) (1, 2). Also, the prevalence of alcohol use is still rising (3). Alcohol abuse is a chronic disease characterized by uncontrolled drinking and preoccupation with alcohol. The patients generally exhibit an inability to control drinking due to both a physical and emotional dependence on alcohol. Notably, it has been indicated that alcohol abuse may cause cerebral aneurysm development and rupture, leading to aneurysmal subarachnoid hemorrhage (aSAH) (4–7). Also, reduced alcohol intake may substantially decrease subarachnoid hemorrhage (SAH) risk (4).

The clinical outcomes following aSAH are associated with multiple factors, including the patient's severity on admission, angiographic vasospasm, and delayed cerebral ischemia (DCI) (8–11). Angiographic vasospasm is the arterial narrowing of large cerebral vessels observed on a radiological test such as CT angiography (CTA), magnetic resonance angiography (MRA), or digital subtraction angiography (DSA) (12). Additionally, angiographic vasospasm is considered a critical factor leading to DCI, which causes poor outcomes or death in up to 30% of patients with SAH (11, 13). While alcohol abuse is a potential risk factor for aSAH, the impact of alcohol abuse on outcomes after aSAH has not been fully evaluated. Herein, we investigated the effect of alcohol abuse on angiographic vasospasm and DCI in a cohort of patients with aSAH requiring mechanical ventilation.

## Methods

### Data Source

We obtained data from the “DATADRYAD” database (https://datadryad.org/search). This website permitted users to freely download the raw dataset. According to Dryad Terms of Service, we cited the Dryad data package in this study [Dryad data package: Chalard et al. (14), Long-term outcome in patients with aSAH requiring mechanical ventilation, Dryad, Dataset, https://doi.org/10.5061/dryad.47d7wm3b4].

### Study Cohort

Chalard et al. completed the entire dataset in the previous study (14). The details were described in the original article. Between January 2010 and December 2015, adult patients with aSAH hospitalized in the neuro-ICU were recruited. Only patients with mechanical ventilation were included in the current study. CTA was performed in all patients at admission to confirm SAH was caused by an aneurysm rupture.

### Patients Characteristics and Clinical Outcomes Collection

The following variables were collected: age, sex, tobacco use, alcohol abuse, diabetes, CVD, Simplified Acute Physiology Score II (SAPSII), World Federation of Neurosurgical Societies (WFNS) score, Fisher score, aneurysm location, and presence of intracerebral hemorrhage (ICH). Type of aneurysm treatment procedure and presence of angiographic vasospasm and DCI were also recorded.

### Statistical Analysis

Continuous variables were expressed as mean ± SD (normal distribution) and categorical variables were expressed in frequency (percentage). The Student's *t*-test (normal distribution) and chi-square test (categorical variables) were used to determine statistical differences between the means and proportions of the groups. Univariate logistic regression analysis was initially performed to identify factors of potential risk, then a multivariable logistic regression model was used to identify independently associated risk factors for the outcomes. The variables included in the multivariable logistic regression analysis were selected on the basis of their associations with the outcomes of interest or a change in effect estimate of more than 10%. All of the analyses were performed using the statistical software packages R (http://www.R-project.org, The R Foundation) and EmpowerStats (http://www.empowerstats.com, X&Y Solutions, Inc., Boston, MA). A *p*-value < 0.05 (two-sided) were considered statistically significant.

## Results

### Patient Demographics and Outcomes

There were 236 patients in this cohort, including 20 patients with alcohol abuse and 216 patients without alcohol abuse. As shown in Table 1, the mean age of non-alcohol abuse patients was 54.87 (SD = 13.32). The mean age of patients with alcohol abuse was 56.25 (SD = 9.94). The comparison of age in the two groups of patients indicated no statistical significance (*p* = 0.651). There were 17 male patients included in the group of alcohol abuse, which was of statistical significance compared with the proportion of alcohol abuse in the female patients (*p* < 0.001). There were 16 patients with tobacco use in alcohol abuse patients with a percentage of 80.00%, which was markedly greater than that in non-alcohol abuse patients (*p* < 0.001). There were 8 patients with diabetes in the non-alcohol abuse group with a percentage of 3.7%. In patients with alcohol abuse, no patients had diabetes. There were 37 patients with CVD in the non-alcohol abuse group (17.13%). In the alcohol abuse group, 3 patients had CVD (15.00%). No statistical significance was observed as to diabetes and CVD (*p* = 0.381, 0.808, respectively) in these two groups.

**Table 1:** Patient demographics and outcomes.

**Alcohol abuse**	**No**	**Yes**	***p*** **-value**
*N*	216	20	
Age	54.87 ± 13.32	56.25 ± 9.94	0.651
Sex			<0.001
Female	144 (66.67%)	3 (15.00%)	
Male	72 (33.33%)	17 (85.00%)	
Tobacco use			<0.001
No	156 (72.22%)	4 (20.00%)	
Yes	60 (27.78%)	16 (80.00%)	
Diabetes			0.381
No	208 (96.30%)	20 (100.00%)	
Yes	8 (3.70%)	0 (0.00%)	
CVD			0.808
No	179 (82.87%)	17 (85.00%)	
Yes	37 (17.13%)	3 (15.00%)	
Aneurysm location			0.882
Ant circ	180 (83.72%)	17 (85.00%)	
Post circ	35 (16.28%)	3 (15.00%)	
SAPSII	42.22 ± 12.09	44.00 ± 11.79	0.528
WFNS score			0.931
III	22 (10.19%)	2 (10.00%)	
IV	88 (40.74%)	9 (45.00%)	
V	106 (49.07%)	9 (45.00%)	
Fisher score			0.807
I	1 (0.46%)	0 (0.00%)	
II	4 (1.85%)	1 (5.00%)	
III	42 (19.44%)	4 (20.00%)	
IV	169 (78.24%)	15 (75.00%)	
ICH			0.721
No	117 (54.17%)	10 (50.00%)	
Yes	99 (45.83%)	10 (50.00%)	
Treatment			0.475
Coil	138 (70.05%)	12 (63.16%)	
Clipping	56 (28.43%)	6 (31.58%)	
Combined	3 (1.52%)	1 (5.26%)	
Angiographic vasospasm			0.058
No	143 (66.20%)	9 (45.00%)	
Yes	73 (33.80%)	11 (55.00%)	
DCI			0.060
No	161 (74.54%)	11 (55.00%)	
Yes	55 (25.46%)	9 (45.00%)	

*CVD, cardiovascular disease; Ant circ, anterior circulation; Post circ, posterior circulation; SAPSII, Simplified Acute Physiology Score II; WFNS, World Federation of Neurosurgical Societies; ICH, intracerebral hemorrhage; DCI, delayed cerebral ischemia*.

As for the location of the aneurysm, there were 180 patients with anterior circulation aneurysms in the non-alcohol abuse group (83.72%). In the alcohol abuse group, 17 patients had anterior circulation aneurysms (85.00%). No statistical significance was detected between the two groups (*p* = 0.882). The mean SAPS II of non-alcohol abuse patients was 42.22 (SD = 12.09). The mean SAPS II of alcohol abuse patients was 44.00 (SD = 11.79). The differences between SAPS II, WFNS score, and Fisher score were of no statistical significance (*p* = 0.528, 0.931, 0.807, respectively). There were 99 patients with ICH in the non-alcohol abuse group (45.83%). In the alcohol abuse group, 10 patients exhibited ICH (50.00%). No statistical significance was detected in the comparison of ICH between the two groups (*p* = 0.721).

A total of 138 patients were treated with a coil and 56 patients were treated with clipping in the non-alcohol abuse group. No statistical significance was observed in the comparison of treatment between the two groups (*p* = 0.475). A total of 73 patients (33.80%) presented with angiographic vasospasm in the non-alcohol abuse group, and 11 patients (55.00%) presented with angiographic vasospasm in the alcohol abuse group, with the *p*-value approaching significance (*p* = 0.058). There were 55 patients (25.46%), who presented with DCI in the non-alcohol abuse group, vs. 9 patients (45.00%) with DCI in the non-alcohol abuse group, a difference that also nearly reached statistical significance (*p* = 0.060).

### Univariate Analysis of Risk Factors for Angiographic Vasospasm and DCI

The risk factors for angiographic vasospasm and DCI were then identified using univariate logistic regression analysis. Alcohol abuse [odds ratio (OR) 2.39, 95% confidence interval (CI) 0.95–6.04; *p* = 0.0643] was likely associated with an increased risk of angiographic vasospasm, with the *p*-value approaching significance. Clipping treatment was associated with a reduced likelihood of angiographic vasospasm (OR 0.26, 95% CI 0.13–0.54; *p* = 0.0003). The results of the univariate analysis of risk factors for angiographic vasospasm are presented in Table 2.

**Table 2:** Univariate logistic regression analysis of risk factors for angiographic vasospasm.

**Variable**	**OR (95% CI)**	***P*** **-value**
Age	0.98 (0.96, 1.00)	0.0613
Sex		
Female	Ref	
Male	0.95 (0.55, 1.64)	0.8492
Tobacco use		
No	Ref	
Yes	1.28 (0.73, 2.25)	0.3913
Alcohol abuse		
No	Ref	
Yes	2.39 (0.95, 6.04)	0.0643
Diabetes		
No	Ref	
Yes	1.09 (0.25, 4.67)	0.9088
CVD		
No	Ref	
Yes	0.47 (0.21, 1.04)	0.0618
Aneurysm location		
Ant circ	Ref	
Post circ	0.69 (0.32, 1.48)	0.3414
SAPSII	0.98 (0.96, 1.00)	0.0460
WFNS score		
III	Ref	
IV	1.02 (0.41, 2.54)	0.9574
V	0.56 (0.23, 1.39)	0.2148
Fisher score		
I	Ref	
II	0.00 (0.00, Inf)	0.9899
III	0.00 (0.00, Inf)	0.9944
IV	0.00 (0.00, Inf)	0.9943
ICH		
No	Ref	
Yes	0.75 (0.44, 1.29)	0.3010
Treatment		
Coil	Ref	
Clipping	0.26 (0.13, 0.54)	0.0003
Combined	3.62 (0.37, 35.58)	0.2702

*CVD, cardiovascular disease; Ant circ, anterior circulation; Post circ, posterior circulation; SAPSII, Simplified Acute Physiology Score II; WFNS, World Federation of Neurosurgical Societies; ICH, intracerebral hemorrhage; ref, reference (OR = 1.0)*.

Alcohol abuse (OR 2.40, 95% CI 0.94–6.09; *p* = 0.0664) was likely associated with increased risk of DCI, with the *p*-value nearly reaching statistical significance. ICH (OR 0.42, 95% CI.23–0.78; *p* = 0.0056) and clipping treatment were associated with a reduced likelihood of DCI (OR 0.19, 95% CI 0.07–0.46; *p* = 0.0003). The results of the univariate analysis of risk factors for DCI are presented in Table 3.

**Table 3:** Univariate logistic regression analysis of risk factors for DCI.

**Variable**	**OR (95% CI)**	***p*** **-value**
Age	0.99 (0.96, 1.01)	0.2141
Sex		
Female	Ref	
Male	1.08 (0.60, 1.95)	0.7940
Tobacco use		
No	Ref	
Yes	0.94 (0.51, 1.75)	0.8484
Alcohol abuse		
No	Ref	
Yes	2.40 (0.94, 6.09)	0.0664
Diabetes		
No	Ref	
Yes	0.37 (0.05, 3.10)	0.3623
CVD		
No	Ref	
Yes	0.42 (0.17, 1.05)	0.0646
Aneurysm location		
Ant circ	Ref	
Post circ	0.55 (0.23, 1.33)	0.1872
SAPSII	0.98 (0.96, 1.00)	0.1136
WFNS score		
III	Ref	
IV	1.03 (0.40, 2.66)	0.9492
V	0.50 (0.19, 1.31)	0.1587
Fisher score		
I	Ref	
II	0.00 (0.00, Inf)	0.9899
III	0.00 (0.00, Inf)	0.9944
IV	0.00 (0.00, Inf)	0.9941
ICH		
No	Ref	
Yes	0.42 (0.23, 0.78)	0.0056
Treatment		
Coil	Ref	
Clipping	0.19 (0.07, 0.46)	0.0003
Combined	0.58 (0.06, 5.67)	0.6362

*CVD, cardiovascular disease; Ant circ, anterior circulation; Post circ, posterior circulation; SAPSII, Simplified Acute Physiology Score II; WFNS, World Federation of Neurosurgical Societies; ICH, intracerebral hemorrhage; ref, reference (OR = 1.0)*.

### Multivariable Analysis of Independent Risk Factors for Angiographic Vasospasm and DCI

The multivariable logistic regression analysis used non-adjusted (crude) and adjusted models to examine the independent risk factors. As shown in Table 4, in model I (adjusted for age and sex), the OR is 2.75, 95% CI 1.03–7.36 (*p* = 0.0440). In model II (adjusted for age, sex, Fisher score, and treatment), the OR is 3.61, 95% CI 1.17–11.17 (*p* = 0.0259). In model III (adjusted for age, sex, Fisher score, treatment, CVD, and SAPS II), the OR is 3.65, 95% CI 1.17–11.39 (*p* = 0.0260). The results of multivariable logistic regression analysis indicated that alcohol abuse was independently associated with increased odds of angiographic vasospasm after adjusting for age, sex, Fisher score, treatment, CVD, and SAPS II.

**Table 4:** Relationship between alcohol abuse and angiographic vasospasm in different models by multivariable logistic regression analysis.

**Exposure**	**Crude**	**Adjust I (OR, 95% CI, *p*)**	**Adjust II (OR, 95% CI, *p*)**	**Adjust III (OR, 95% CI, *p*)**
Alcohol abuse				
No	1.0	-	-	-
Yes	2.39 (0.95, 6.04) 0.0643	2.75 (1.03, 7.36) 0.0440	3.61 (1.17, 11.17) 0.0259	3.65 (1.17, 11.39) 0.0260

*Crude model: we did not adjust other variables*.

*Adjust I: we adjusted age and sex*.

*Adjust II: we adjusted age, sex, Fisher score, and treatment*.

*Adjust III: we adjusted age, sex, Fisher score, treatment, CVD, and SAPS II*.

*CVD, cardiovascular disease; SAPSII, Simplified Acute Physiology Score II*.

Similarly, the ORs of alcohol abuse for DCI were calculated in different adjusted models (Table 5). The results suggested that alcohol abuse was independently associated with increased odds of DCI (OR 3.53, 95% CI 1.13–10.97; *p* = 0.0294, model III).

**Table 5:** Relationship between alcohol abuse and DCI in different models by multivariable logistic regression analysis.

**Exposure**	**Crude**	**Adjust I (OR, 95% CI, *p*)**	**Adjust II (OR, 95% CI, *p*)**	**Adjust III (OR, 95% CI, *p*)**
Alcohol abuse				
No	1.0	-	-	-
Yes	2.40 (0.94, 6.09) 0.0664	2.54 (0.94, 6.87) 0.0656	3.52 (1.14, 10.85) 0.0285	3.53 (1.13, 10.97) 0.0294

*Crude model: we did not adjust other variables*.

*Adjust I: we adjusted age and sex*.

*Adjust II: we adjusted age, sex, Fisher score, and treatment*.

*Adjust III: we adjusted age, sex, Fisher score, treatment, CVD, and SAPS II*.

*DCI, delayed cerebral ischemia; CVD, cardiovascular disease; SAPSII, Simplified Acute Physiology Score II*.

## Discussion

Angiographic vasospasm and DCI are strongly associated with the outcomes following aSAH (11, 13). This study aims to have a better understanding of the risk factors involved that may lead to angiographic vasospasm and DCI. Alcohol abuse has about 2.5-fold higher odds of angiographic vasospasm and DCI *via* multivariable logistic regression analysis when adjusting for other potential confounding variables. Our findings provide important evidence for the prevention and clinical management of aSAH.

Although previous studies have suggested that alcohol abuse increases the risk of aSAH (4), the role of alcohol abuse in the development of angiographic vasospasm and DCI has not been fully investigated yet. Because alcohol abuse is present in a large proportion of patients with aSAH, especially in men, identification of alcohol abuse as an independent risk factor of angiographic vasospasm and DCI is of great clinical significance in determining how closely the patients should be monitored for these two events.

Alcohol has been suggested to induce vessel constriction *via* various mechanisms. Alcohol can increase the activity of the sympathetic nervous system and the release of catecholamines, leading to the constriction of blood vessels (15, 16). Besides, alcohol has been indicated to decrease the levels of electrically charged (i.e., ionized) magnesium in plasma (17). A delicate balance between magnesium and calcium ions is needed to maintain vascular tone at a normal level. Magnesium triggers vessel relaxation and calcium, on the contrary, causes vessel constriction. When the level of magnesium ions is reduced by alcohol, the calcium ions will predominate, resulting in vessel constriction. These mechanisms help to explain alcohol abuse as an independent risk factor for angiographic vasospasm after aSAH in this study.

Apart from the effect on vascular tone, alcohol may lead to endothelial dysfunction. A high dose of alcohol consumption leads to an increased endothelin level, which is also involved in vessel constriction (18, 19). Alcohol abuse also leads to oxidative stress. For example, a high dose of alcohol leads to the overproduction of reactive oxygen species, leading to the peroxidation of lipids, proteins, and DNA and ultimately to necrosis and apoptosis (20). The increased level of endothelin induced by alcohol abuse is also associated with oxidative stress (18). The blockade of oxidative stress may prevent endothelial dysfunctions induced by alcohol. A study by Sacanella et al. indicated that alcohol abuse increases the expression of intercellular adhesion molecule (ICAM-1) and E-selectin, which participates in the adhesion and migration of inflammatory cells. As for inflammation, a high dose of alcohol consumption may cause an increase in tumor necrosis factor-alpha (TNF-alpha) and interleukin-6 (IL-6) (21), contributing to the aggregation of inflammatory cells and vessel constriction or spasm.

Alcohol also acts on coagulation and fibrinolysis. It has been demonstrated that heavy alcohol intake is associated with lower fibrinolytic capacity and a more procoagulant state, with an elevation in the plasma levels of factor VII, fibrinogen, and viscosity (22). Fibrinolysis is mainly regulated by two proteins in the blood: tissue plasminogen activator (tPA) and plasminogen activator inhibitor 1 (PAI-1). TPA promotes fibrinolysis, whereas PAI-1 inhibits fibrinolytic activity. Heavy alcohol consumption has been indicated to stimulate PAI-1 production and thereby suppress fibrinolysis (23). Fibrinolysis suppression may lead to subsequent thrombosis and vessel spasms.

Angiographic vasospasm has been indicated to be present in up to 70% of patients following SAH (9), and it has been regarded as an important factor causing DCI for a long time. However, recent findings have suggested that angiographic vasospasm alone is not sufficient to trigger DCI (9). DCI is only found in about 30% of patients and does not always fall within the vascular distribution of the angiographic vasospasm (24). DCI, therefore, may occur in an area that does not involve angiographic vasospasm. In our study, DCI was found in 27% of patients, which is nearly 30%. For the non-alcohol abuse group, 73 patients presented with angiographic vasospasm and 55 with DCI. For the alcohol abuse group, 11 with angiographic vasospasm, and 9 with DCI. Notably, the patients with DCI all presented with the presence of angiographic vasospasm, which verified the critical role of angiographic vasospasm in the development of DCI following aSAH. However, further research is still needed to find out other causes of DCI.

### Limitations

There are some limitations to our study. First, this is a single-center study based on a French patients cohort, and only patients with mechanical ventilation were analyzed. Therefore, the generalizability of the results to other geographical areas and the patient groups may be limited. Second, this is a retrospective study, so we can only conclude that alcohol abuse was associated with angiographic vasospasm and DCI, but causation cannot be proved. Therefore, only an OR could be calculated, not the relative risk. Third, due to raw data limitations, the dose of alcohol consumption was not recorded, which would otherwise provide more details regarding the effect of alcohol on angiographic vasospasm and DCI.

## Conclusions

In this study, we have demonstrated that patients with alcohol abuse havehas about 2.5-fold higher odds of angiographic vasospasm and DCI compared to non-alcohol abuse patients after aSAH, independent of age, sex, Fisher score, treatment, CVD, and SAPS II. Our findings may be helpful in monitoring patients with known risk factors for the development of angiographic vasospasm and DCI following aSAH.

## Data Availability Statement

The dataset is available on Dryad at DOI: 10.5061/dryad.47d7wm3b4.

## Ethics Statement

The studies involving human participants were reviewed and approved by Comite de Protection des Personnes Sud-Mediterannee IV, Montpellier, France; ID: Q-2015-09-07. Written informed consent for participation was not required for this study in accordance with the national legislation and the institutional requirements.

## Author Contributions

LZhao wrote the first draft of the manuscript. All authors contributed to the conception and design of the study, statistical analysis, manuscript revision, read, and approved the submitted version.

## Conflict of Interest

The authors declare that the research was conducted in the absence of any commercial or financial relationships that could be construed as a potential conflict of interest.

## Publisher's Note

All claims expressed in this article are solely those of the authors and do not necessarily represent those of their affiliated organizations, or those of the publisher, the editors and the reviewers. Any product that may be evaluated in this article, or claim that may be made by its manufacturer, is not guaranteed or endorsed by the publisher.
